# Fused Filament Fabrication (FFF) for Manufacturing of Microfluidic Micromixers: An Experimental Study on the Effect of Process Variables in Printed Microfluidic Micromixers

**DOI:** 10.3390/mi12080858

**Published:** 2021-07-22

**Authors:** Mojtaba Zeraatkar, Marco D. de Tullio, Gianluca Percoco

**Affiliations:** Department of Mechanics, Mathematics and Management, Polytechnic University of Bari, 70126 Bari, Italy; marcodonato.detullio@poliba.it (M.D.d.T.); gianluca.percoco@poliba.it (G.P.)

**Keywords:** additive manufacturing, FFF printing, micromixer, microfluidics

## Abstract

The need for accessible and inexpensive microfluidic devices requires new manufacturing methods and materials as a replacement for traditional soft lithography and polydimethylsiloxane (PDMS). Recently, with the advent of modern additive manufacturing (AM) techniques, 3D printing has attracted attention for its use in the fabrication of microfluidic devices and due to its automated, assembly-free 3D fabrication, rapidly decreasing cost, and fast-improving resolution and throughput. Here, fused filament fabrication (FFF) 3D printing was used to create microfluidic micromixers and enhance the mixing process, which has been identified as a challenge in microfluidic devices. A design of experiment (DoE) was performed on the effects of studied parameters in devices that were printed by FFF. The results of the colorimetric approach showed the effects of different parameters on the mixing process and on the enhancement of the mixing performance in printed devices. The presence of the geometrical features on the microchannels can act as ridges due to the nature of the FFF process. In comparison to passive and active methods, no complexity was added in the fabrication process, and the ridges are an inherent property of the FFF process.

## 1. Introduction

Microfluidics is an emerging field with a large number of chemical and biological applications that have been developed over the last few years, including biomedical diagnostics, DNA and protein analysis, food safety control, and drug development [1,2,3]. Given the microscale dimensions, fluidic behavior in microfluidic devices is characterized by low values of Reynolds numbers (Re < 100) that result in laminar flow. Under laminar flow, mixing between two or more adjacent parallel fluid streams is slow and inefficient because diffusion is the only mixing mechanism. Therefore, long channels are required to achieve complete mixing [4]. Many types of attractive applications, such as membraneless fuel cells [5,6], DNA [2,7] and sorting (of cells) [8,9] have exploited laminar flow in microscale devices. There are also many applications, such as biochemical assays [10,11,12] and chemical reactions [13,14,15,16], where rapid mixing and complete mixing of solutions are required.

In the past two decades, various systems for achieving complete mixing within a reasonable timeframe and length of microchannel have been introduced. These systems are generally divided into passive and active, with a full discussion provided in several relevant literature reviews [4,17,18,19]. Passive micromixers do not require an external energy source except the energy used to drive the fluids. In these systems, an attempt has been made to enhance the diffusion or chaotic advection by altering the structure or configuration of channels such as zigzag and split-and-recombine (SAR) micromixers [20]. In contrast, active micromixers utilize external energy sources such as pressure [21], temperature [22], electrokinetics [23], magnetics [24], acoustics [25], or a mechanical actuation mechanism for disturbing the fluids, and therefore increasing the contact area. While each method has its own advantages and disadvantages, the fabrication process of the microfluidic micromixers is also essential. Passive and active methods typically require complex geometries of ridges or grooves and need to have movable parts, which increase the difficulty for fabrication. In the past two decades, the vast majority of microfluidic micromixers have been built in poly dimethylsiloxane (PDMS) by soft-lithography, a process based on PDMS micro-molding that demands specialized operators, costly procedures, and dedicated infrastructure such as clean rooms [26].

The advent of modern additive manufacturing (AM) techniques has enabled the transfer of most of the fabrication skills to 3D printers as a potential replacement for rapid prototyping through soft lithography due to its automated, assembly-free 3D fabrication, rapidly decreasing cost, and fast-improving resolution and throughput [27]. Among the different 3D printing methods, those most relevant to microfluidic device fabrication are stereo lithography (SLA), multijet modeling (MJM), and fused filament fabrication (FFF) [28]. Several groups, including ours, have experimentally compared the advantages and disadvantages of each method for use in microfluidics [29,30]. FFF is one of the many types of AM processes in which a heated nozzle is used for the extrusion of a polymer filament in 2D layers to form the desired 3D object [31]. It is a clean and inexpensive process with a large selection of biocompatible materials and there is no need for liquid components or post-processing. The polymers used in FFF owe their success to four fundamental properties: biocompatibility, transparency, low cost, and being copyright-free [28]. Due to continual progress being made in the development of the FFF technique, the printing of transparent microfluidic devices with channel dimensions consistently under 100 μm, formerly exclusive to SLA, can now also be obtained with FFF [32,33], which bring the benefit of precisely defined printing materials.

Thanks to the popularity of surface patterning in the creation of passive micromixers, FFF can be a simple and effective way to fabricate micromixers. Several studies have demonstrated the effect of patterned grooves or ridges on fluidic behavior in microchannels [34,35,36,37,38,39,40]. Stroock et al. [34] developed one of the first passive micromixers by placing two different groove patterns and showed that mixing could be enhanced in a staggered herringbone mixer (SHM) at a Reynolds number from 1 to 100. Johnson et al. [36] developed a slanted groove mixer (SGM) using an excimer laser system to create a series of slanted wells on the microchannel floor to achieve quick mixing by introducing transverse transportation of the fluids. Many other different types of patterned grooves similar to those by Johnson et al. and Stroock et al. have been studied to show the effects of various micro and macro ridges or grooves on mixing enhancement in microfluidic devices [38,40,41]. Yun et al. [42] investigated the geometric effects of fabricated grooves using laser cutting and milling machines on the cross-movement of dye solutions. The results showed the effect of groove depth on the lateral transport of dye solutions. Kwak et al. [39] studied the mixing efficiencies of negative and positive groove patterns. Their study showed that a positive pattern has a better mixing efficiency than the conventionally used negative pattern. There are limited works that have used the inherent and unique features of FFF printers to make passive ridge-based micromixers. Li et al. [43] used the extrusion orientation for enhancing the mixing behavior in microfluidic chips made by FFF and showed that the fluidic devices that were printed with the extruded filament at 60° relative to the flow had the highest mixing efficiency.

Here, the effects of process variables in FFF process study on the fluidic behavior inside microchannels; with a focusing on the surface roughness of the FFF method which is introduced as a limitation of the process. The effects of extruded filament, material transparency, flow rate, and machine study on the devices printed by FFF. A design of experiment was performed on the effects of different parameters, and the experimental results are analyzed using a colorimetric approach. We use the limitations of FFF as an attractive tool for the enhancement of mixing behavior in microfluidic micromixers, together with a reduction in the complexity of the fabrication process that is identified in most microfluidic micromixers.

## 2. Materials and Methods

### 2.1. Materials

Two different types of polylactic acid (PLA) filament with a 2.85 mm diameter were purchased from Fabbrix, Italy: (i) transparent PLA and (ii) translucent PLA. PLA is the most commonly used biocompatible polymer in drug delivery systems, tissue engineering, and temporary and long-term implantable devices [44]. In addition to biocompatibility, transparency of the printed device is a vital point in microfluidics. Transparency allows fluid interactions to be clearly visible through the detection system.

Two water streams were introduced into the Y-shaped microfluidic micromixers, with yellow and blue food dyes (purchased from a local shop) used to distinguish one stream from the other. In micromixer studies, flow and transport characteristics can be defined through two dimensionless numbers, i.e., the Reynolds (Re) number and Peclet (Pe) number [45], as shown in Equation (1), respectively:

(1) Re=Deq×Vv; Pe=Deq×VD

where $D_{eq}$ is the hydraulic diameter of the channel and defined as 4A/P_w_; where A is the cross-sectional area; P_w_ is the wetted perimeter of the channel at the cross-section; V indicates the fluid velocity; v is the kinematic viscosity; and *D* is the diffusion coefficient. In this study, the experimental tests were carried out at two different flow rates with Reynolds numbers, Re = 2.2 and Re = 8.87. The corresponding Peclet numbers are 2.2 × 10^4^ and 8.87 × 10^4^, respectively, with diffusion coefficient in the order of 1 × 10^−10^ m^2^ s^−1^.

### 2.2. Instrumentation

**Figure 1:**
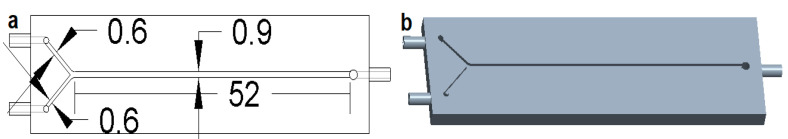
CAD illustrations of the micromixer design. (**a**) CAD drawing of the designed micromixer with dimensions in mm; (**b**) 3D CAD model of the micromixer.

**Figure 2:**
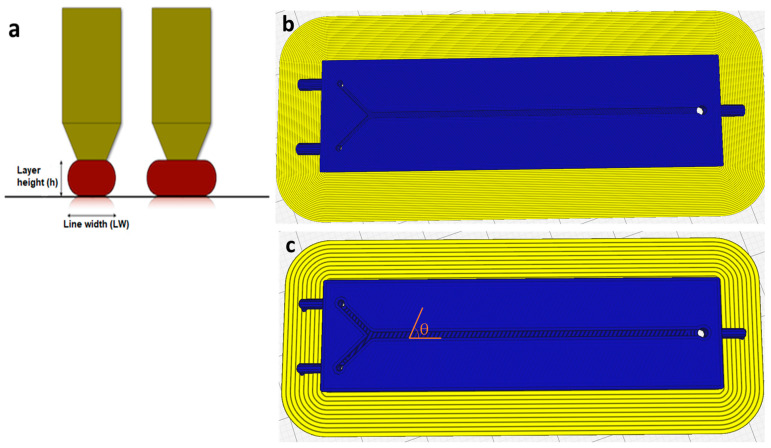
Printing parameters: (**a**) the geometry of the extruded filament; (**b**,**c**) show the movement path of the printing nozzles with two different infill line widths, 200 and 600 µm, respectively. The angle θ denotes the orientation of the extruded filament relative to the fluid flow in the channel.

The microfluidic micromixers were designed using PTC Creo (Student Edition) and exported in STL format. Figure 1 shows a simple Y-shaped micromixer with two inlets and one outlet, which was used in this study. The mixing channel was 52-mm-clong (from the Y-junction); the width and depth of the main channel were 900 and 600 μm, respectively. The devices were printed using two different printers, the Ultimaker 3 and the Ultimaker S5 (Ultimaker, Geldermalsen, The Netherlands). The declared layer resolution is 20–200 microns in the Ultimaker 3, and 60–150 and 20–200 microns in the Ultimaker S5 for 0.25 mm and 0.4 mm nozzles, respectively. The accuracy (XYZ) was 12.5, 12.5, and 2.5, and 6.9, 6.9, and 2.5 microns for the Ultimaker 3 and Ultimaker S5 printers, respectively. Two different nozzles were used for printing microchips: a 0.25 mm nozzle for devices fabricated with a line width of 200 µm, and a 0.4 mm nozzle for devices with a line width of 600 µm. The open-source Ultimaker Cura 4.8.0 software used for setting printing parameters and for slicing .stl files into G-code. For all devices, the layer height was set to 100 μm, and the filament orientation was adjusted at 60° relative to the fluid flow (Figure 2a,c), which resulted in a better mixing performance [43]. Table 1 shows the fixed printing parameters that were set for the two nozzles during all the experiments. A Nikon Eclipse MA200 microscope was used for taking photographs from the printed microchannels (Nikon Corporation, Tokyo, Japan).

### 2.3. Design of Experiments (DoE)

In the literature, several factors have been noted that can be used to model the surface profile and the resulting surface roughness in the printing process, such as the cross-sectional shape of the deposited filaments, infill line width, infill orientation, and layer thickness (Figure 2) [46]. Additionally, the overlap distance between adjacent layers and the distance between adjacent filaments, which exist in the vertical and horizontal directions, can act as important parameters for the surface roughness of printed devices. For applications such as microfluidics, the effect of each parameter should be considered with regard to the velocity of fluids. It should be noted that the resolution of 3D printer can also have a direct impact on the resulting surface roughness. The channel surface of a device printed with a high-resolution printer is smoother than devices printed with a low-resolution printer. There are few studies on the effect of the surface roughness present in FFF on fluidic behavior in microfluidics. The effect of infill orientation on fluidic behavior was studied by Li et al. [43]. In this study, the process variables are infill line width, machine (3D printer), material transparency, and flow rate. The response variable is the required length for the complete mixing.

**Figure 3:**
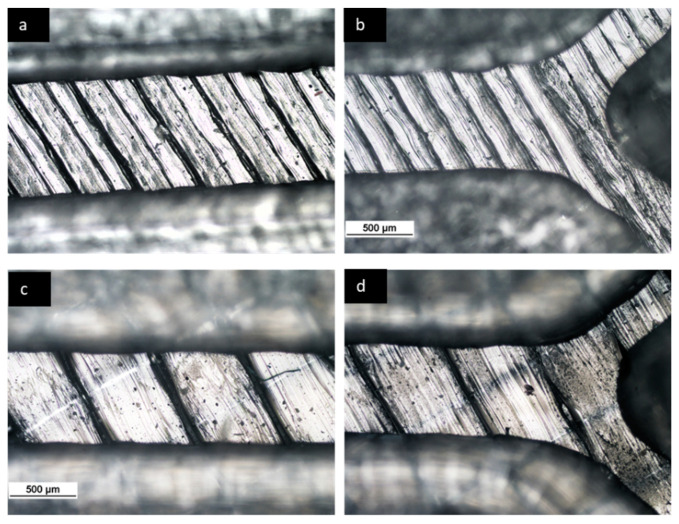
Photographs of different fabricated channels: (**a**,**b**) show the fabricated device with a line width of 200 µm; (**c**,**d**) show the fabricated device with a line width of 600 µm.

A full factorial design (2^4^) with two replications was performed for each combination. In order to find the best levels, four devices with line widths of 0.2, 0.4, 0.6, and 0.8 mm were fabricated first by keeping the other printing parameters constant (see Table 1). The fabricated devices with a line width greater than 0.6 mm showed leakage at flow rates higher than 200 µL/min. Therefore, 0.2 mm and 0.6 mm were selected as the low and high levels of the line width parameter (Figure 2b,c). The infill line distance (horizontal gap) for devices printed with a 0.25 mm nozzle and a 0.4 mm nozzle was set to 0.2 mm and 0.6 mm, respectively, meaning that the two adjacent filaments were tangent to each other. However, due to the shrinkages and the resolution of the process, a gap between the adjacent filaments was observed for all fabricated devices (Figure 3). Flow rates of 50 and 100 µL/min were selected as the two levels (low to medium fluid velocities) for the flow rate factor. Several groups, including ours, showed that the FFF-printed micromixers have the same mixing performance as mixers printed by other 3D printing methods at higher flow rates [29,30]. The factors and the respective levels are summarized in Table 2.

### 2.4. Experimental Setup

**Figure 4:**
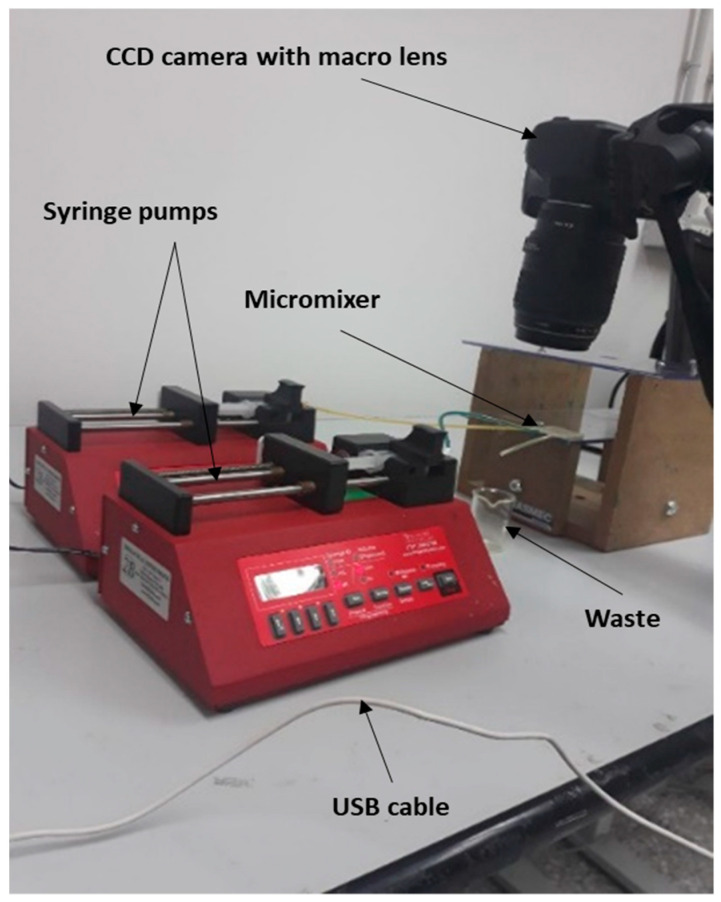
Experimental setup and detection system for testing our microfluidic systems.

The performance of the fabricated micromixers in mixing two solutions was tested using the experimental setup shown in Figure 4. First, all devices were cleaned with water and dried using warm air. Compressed air jets were used to eliminate any drops of water trapped inside the microchannels. Tape (3M Ruban Adhesif Scotch^®^) was used to seal the top surfaces, thus closing the open sides of the microfluidic micromixers. Two different water streams colored yellow and blue were injected using two syringe pumps (Braintree, MA, USA, Mod. BS-300) equipped with 5-mL disposable syringes at flow rates of 50 and 100 μL/min. The streams were precisely controlled, and the flow rates of both inlets were set equally (the total flow rates in the main channel were 100 and 200 μL/min). After achieving steady-state conditions, the fluid within the microchannels was captured using a CCD camera (Canon EOS 4000D, Tokyo, Japan) with a macro lens (Canon EF 100 mm f/2.8 Macro USM, Tokyo, Japan). The captured images were then sent to a server using a USB cable for further processing by a MATLAB algorithm (see Supplementary Materials).

## 3. Results and Discussion

**Figure 5:**
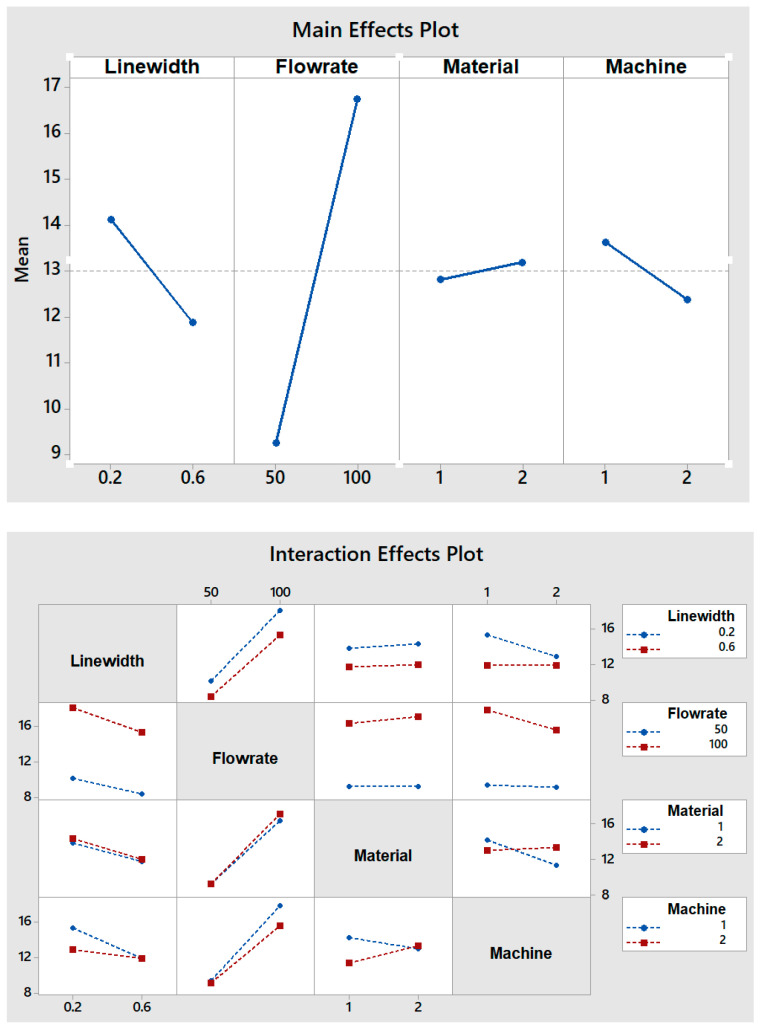
Main and interaction effects plots for the length of mixing.

In Table 3, the analyzed results of the different combinations in the full factorial design are summarized. The results are the performance of the FFF-printed micromixers at two different Reynolds numbers (Re = 2.2 and Re = 8.87 for flow rates of 50 and 100 µL/min, respectively). The results show the effects of different factors and levels on the required channel length for achieving complete mixing. In Figure 5, the main and interaction effects for the length of mixing are shown. Achieving complete mixing over a short time and length of channel is one of the main challenges in microfluidic micromixers.

**Figure 6:**
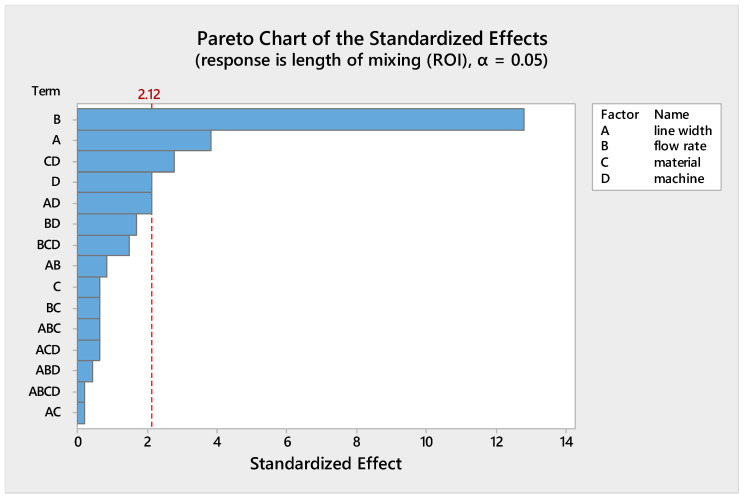
Pareto chart of standardized effects.

**Figure 7:**
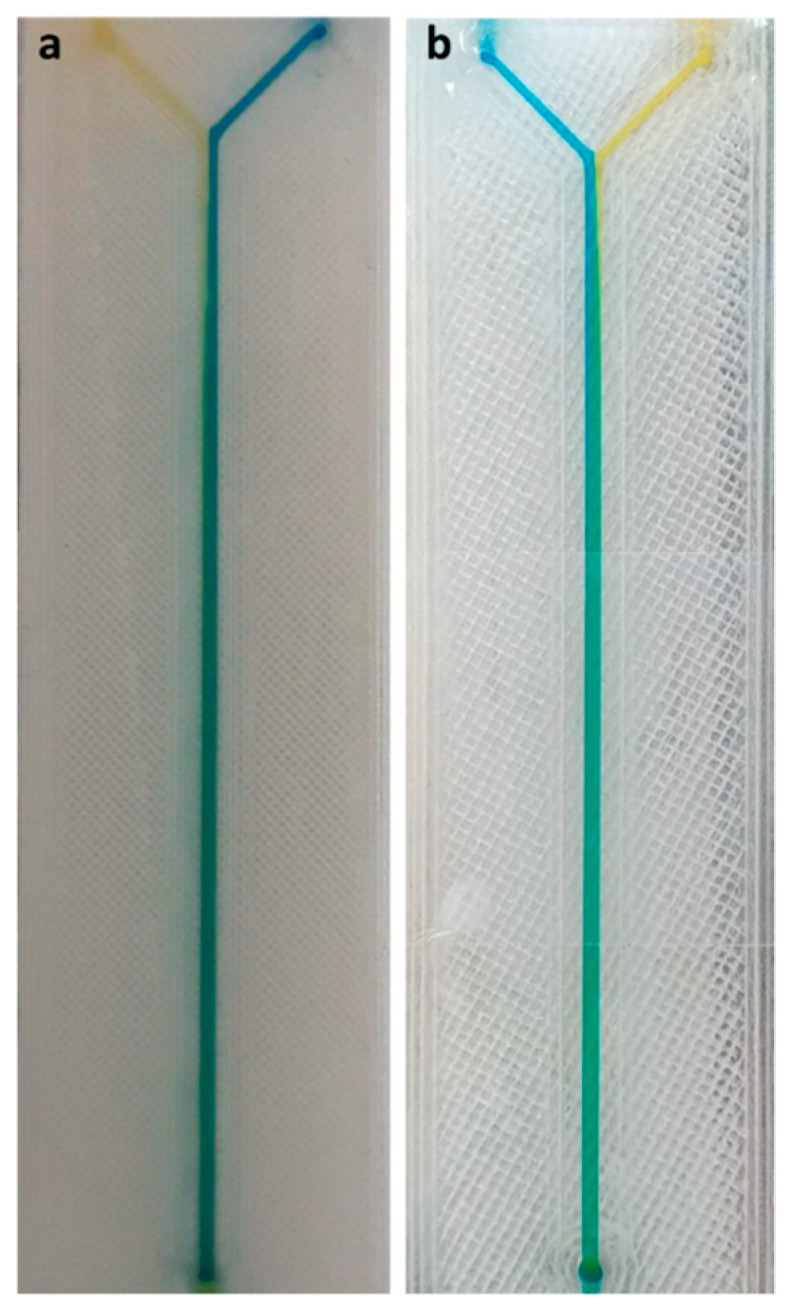
Full top view of 3D printed microfluidic devices, demonstrating transparency: (**a**) translucent PLA; (**b**) transparent PLA.

Several results can be drawn from plots of the main and interaction effects:

(1) The flow rate is the factor with the greatest effect on the length of mixing. With an increase in the flow rate from 50 to 100 µL/min, the required length for complete mixing was increased in all fabricated micromixers, which is consistent with other studies [29,30]. As shown in the Pareto chart and Table 4, the interaction effects of this factor are insignificant. Figure 6 shows the Pareto chart of the standardized effects, while in Table 4, the effects (non-standardized) and respective T-values and *p*-values (α = 0.05) for each factor and their interaction effects are summarized.
(2) Fabricated micromixers with an extrusion line width of 600 µm showed a better mixing performance than devices printed with a line width of 200 µm, where the average required length for complete mixing is decreased. However, the interaction effects of line width are negligible (see Figure 6 and Table 4).
(3) The effect of material on the length of mixing is negligible due to the equal level of hydrophobicity for the materials used. The surface of PLA is strongly hydrophobic [47], and the use of hydrophobic surfaces is one of the topics of interest for mixing enhancement in microfluidic systems [48,49]. Although the effect of the material was insignificant on the mixing process, the transparency of printed devices is another vital point in the fabrication of microfluidic devices. Examples of the printed micromixers with translucent and transparent PLA are shown in Figure 7. The devices printed with translucent PLA were often opaque, rendering optical analysis challenging. The semi-transparency of the printed device with the transparent PLA allows imaging with back illumination [29].
(4) Using different printers can cause different results due to the different accuracy of the printers. The two printers used in this study had a slight effect on the required length of mixing (see Figure 5). In general, the printed channels in the FFF process are consistently smaller than the CAD model, due to the spreading of the polymer as it is extruded [30]. For this, the mixers printed with the Ultimaker S5 had a better mixing performance, as its higher accuracy allowed the printing channels to be closer to the designed model. Among the different interaction effects, the effect of C*D (material*machine) is significant (see Table 4). A probable explanation is related to the ability of the machines to print different materials. While the results for the printed mixers with the translucent PLA were somewhat similar in two printers, the fabricated mixers with the transparent PLA had different performances when printed with two different printers (see interaction effects plot in Figure 5).

FFF is a technique in which a filament is heated and deposited layer by layer based on a generated section data from a CAD model. The typical roughness, due to the nature of the process itself, on the FFF channel surface can act as ridges (see Figure 3). The ridges on the bottom of the channels can affect the fluidic behavior and enhance the “poor” mixing performance, which is identified as a challenge in microfluidic micromixers. In this study, the devices with a greater extruded line width (600 µm) showed better mixing efficiency, which can be explained by considering the transverse movement of fluids induced by obliquely patterned ridges that are the result of the extruded filaments in FFF. The lateral displacements generate a helical flow along the axis of flow and enhance mixing efficiency, as described in [34,38]. However, in the devices printed with an extruded line width equal to 200 µm, a longer length of channel is needed for complete mixing, due to a small gap between two ridges (see Figure 3). Inside a single groove, the lateral displacements expand by increasing the gap between two ridges; however, the results of our experiment showed the existence of leakage in printed micromixers with an infill line distance greater than 600 µm. It should also be noted that a deeper groove can result in a larger dead volume, which makes mixing inefficient where solutions can be retained for extremely long periods in real applications [50].

These experimental observations are consistent with the effect of groove patterns that was found for the first time in SGM and SHM passive micromixers [34,36], with this difference being that the ridges in the FFF method are an inherent property of the process and do not increase the complexity of the fabrication process. In this study, the examined flow rates represented the low-to-medium flow rates for each stream. The effect of extruded filaments on fluidic behavior should be considered with respect to inlet velocities. At higher flow rates, our previous results showed a slight change in the required length of mixing between FFF-printed micromixers and devices printed by other methods (SLA, Polyjet) [29]. This can be explained by the absence of such cross-movements at high flow rates inside the grooves, where the fluids remain inside the grooves and form a dead volume.

**Figure 8:**
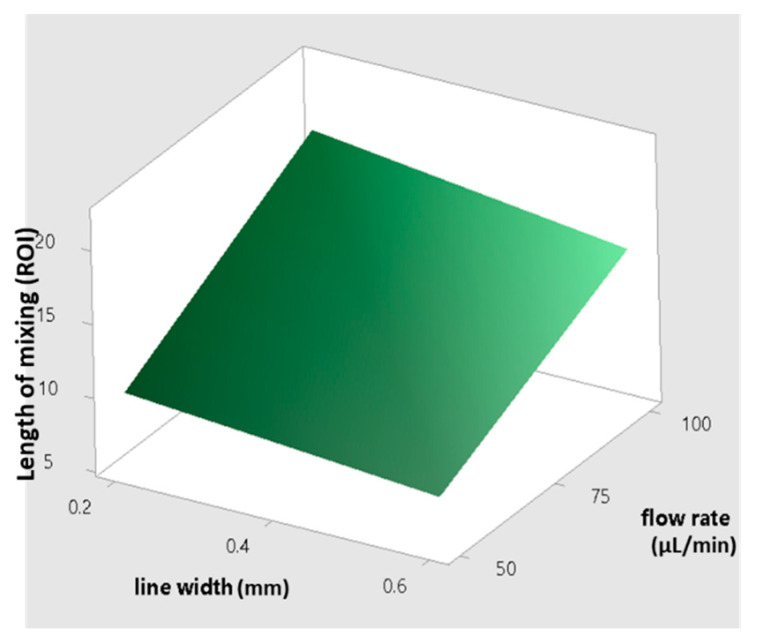
Surface plot for predicting the length of mixing versus different line width and flow rates.

A model for predicting the required channel length for complete mixing with regard to the extruded line width and flow rate is shown in Figure 8. The presence of typical roughness on the bottom surface of the channels makes FFF an attractive tool for applications where mixing two or more fluids in a short time and length of channel is required. The enhancement of the mixing performance leads to a reduction in the size of the mixer, which is very important when complex reactions must be achieved in a single device. As an example of a possible application, a new trend in commercial molecular biology could be considered: standard platforms for both centralized and decentralized testing locations [51,52]. These systems are based on cartridge technology, allowing for changes in testing capabilities depending on the cartridge used. Different tests need cartridges with different microfluidic channels. The size of the cartridge must be the same for all the possible molecular tests. The use of FFF can reduce the length of the channel, helping to keep the size of the device and the cartridge within the specified bounding box for that platform, thus keeping costs low.

## 4. Conclusions

We demonstrated here that FFF, an inexpensive and automated printing method with a large selection of materials, can be a viable technique for manufacturing microfluidic micromixers. The effects of extruded filament, flow rate, material transparency, and 3D printer on devices fabricated by FFF has been studied. The devices that were printed with a line width of 0.6 mm and with the Ultimaker S5 printer showed a better mixing performance, especially at low flow rates (50 µL/min). The use of extruded filament in devices printed by FFF can result in ridges that increases the mixing performance within the microchannels, similar to groove-patterned micromixers that are fabricated by other methods. The existing methods typically enhance mixing by exploiting the complex geometry of the channels (passive methods) or by using movable parts (active methods); while in FFF, the presence of the geometrical features on the microchannels is due to the nature of the process and does not increase the complexity of the fabrication process. The results show the effect of a high extruded width on the mixing process; however, choosing line widths greater than 600 μm leads to leakage at higher flow rates (200 µL/min), and it should also be noted that the rough surface that is created may be a drawback for applications where a smooth channel surface is required.

## Figures and Tables

**Table 1:** Fixed printing parameters during the experiments.

Printing Parameter	Nozzle 0.25 mm	Nozzle 0.4 mm
Printing temperature (°C)	190	200
Printing speed (mm/s)	30	70
Layer height (μm)	100	100
Filament orientation (°)	60	60
Flow (%)	100	100
Infill line distance (mm)	0.2	0.6
Build temperature (°C)	60	60

**Table 2:** Factors and levels.

Factor	Level	
	−1	+1
Line width (LW)	200 μm	600 μm
Flow rate	50 µL/min	100 µL/min
Material	Transparent PLA	Translucent PLA
Machine	Ultimaker 3	Ultimaker S5

**Table 3:** The performance of mixing in each FFF printed micromixer, evaluated by colorimetric analysis.

Run Number	A (Line Width)	B (Flow Rate)	C (Material)	D (Machine)	Run Label	Length of Mixing (ROI)	
						n_1_	n_2_
1	−1	−1	−1	−1	(1)	12	10
2	+1	−1	−1	−1	a	10	7
3	−1	+1	−1	−1	b	22	19
4	+1	+1	−1	−1	ab	18	16
5	−1	−1	+1	−1	c	11	10
6	+1	−1	+1	−1	ac	8	7
7	−1	+1	+1	−1	bc	21	18
8	+1	+1	+1	−1	abc	16	13
9	−1	−1	−1	+1	d	9	10
10	+1	−1	−1	+1	ad	7	9
11	−1	+1	−1	+1	bd	13	16
12	+1	+1	−1	+1	abd	14	13
13	−1	−1	+1	+1	cd	9	10
14	+1	−1	+1	+1	acd	9	10
15	−1	+1	+1	+1	bcd	20	16
16	+1	+1	+1	+1	abcd	15	18

**Table 4:** Non-standardized effects, T- and *p*-values in factorial design.

Factor/*n* Way Interactions	Effect	T-Value	*p*-Value
A	−2.25	−3.84	0.001
B	7.50	12.79	0.000
C	0.375	0.64	0.531
D	−1.25	−2.13	0.049
A*B	−0.50	−0.85	0.406
A*C	−0.125	−0.21	0.834
A*D	1.25	2.13	0.049
B*C	0.375	0.64	0.531
B*D	−1.00	−1.71	0.107
C*D	1.625	2.77	0.014
A*B*C	−0.375	−0.64	0.531
A*B*D	0.25	0.43	0.675
A*C*D	0.375	0.64	0.531
B*C*D	0.875	1.49	0.155
A*B*C*D	−0.125	−0.21	0.834

## Data Availability

Data is contained within supplementary material.

## Supplementary Materials

The following are available online at https://www.mdpi.com/article/10.3390/mi12080858/s1, Figure S1: Data flowchart of the colorimetric analysis, Figure S2: (a) Captured image from the top of the device; (b) detection of the main channel from the input image; (c) different ROIs along the main channel at regular intervals, Figure S3: Mixing trend diagrams at flow rates of 50 and 100 µL/min. At the beginning of the main channel, as the fluids are not yet mixed, the curves have two peaks. The curve corresponding to the ROI with complete mixing is a straight line, Figure S4: Normalized mean square errors (MSEs) versus different ROIs at flow rates of 50 and 100 µL/min. Complete mixing is achieved after ROI number 7 and 16 at flow rates of 50 and 100 µL/min, respectively.
