# Supplementary material for: Fused Filament Fabrication (FFF) for Manufacturing of Microfluidic Micromixers: An Experimental Study on the Effect of Process Variables in Printed Microfluidic Micromixers
Source: Micromachines (Basel). 2021 Jul 22;12(8):858. doi: 10.3390/mi12080858 (PMC8399612; doi:10.3390/mi12080858)
Supplement: Supplementary file 1 [file micromachines-12-00858-s001.zip › micromachines-1278480-supplementary.pdf]

Colorimetric Analysis

The performance of the printed devices in mixing the two fluids was evaluated by analyzing images taken from the microchannels. The flowchart of the colorimetric analysis is shown in Figure S1. The first step in the colorimetric analysis was the extraction of the main channel from the image background to determine a set of regions of interest (ROIs). Figure S2b shows the extracted microchannel at a grayscale level. The next step considered a set of 40 rectangular ROIs at regular intervals (the distance between each ROI was 1.3 mm) perpendicular to the main channel to obtain the change in color intensity across the channel (Figure S2c). For every ROI covering the width of the channel, the obtained intensity values were normalized to the intensity of the dyed streams before mixing, the pixels were averaged, and the standard deviation was calculated for every position along w (width of single ROI).

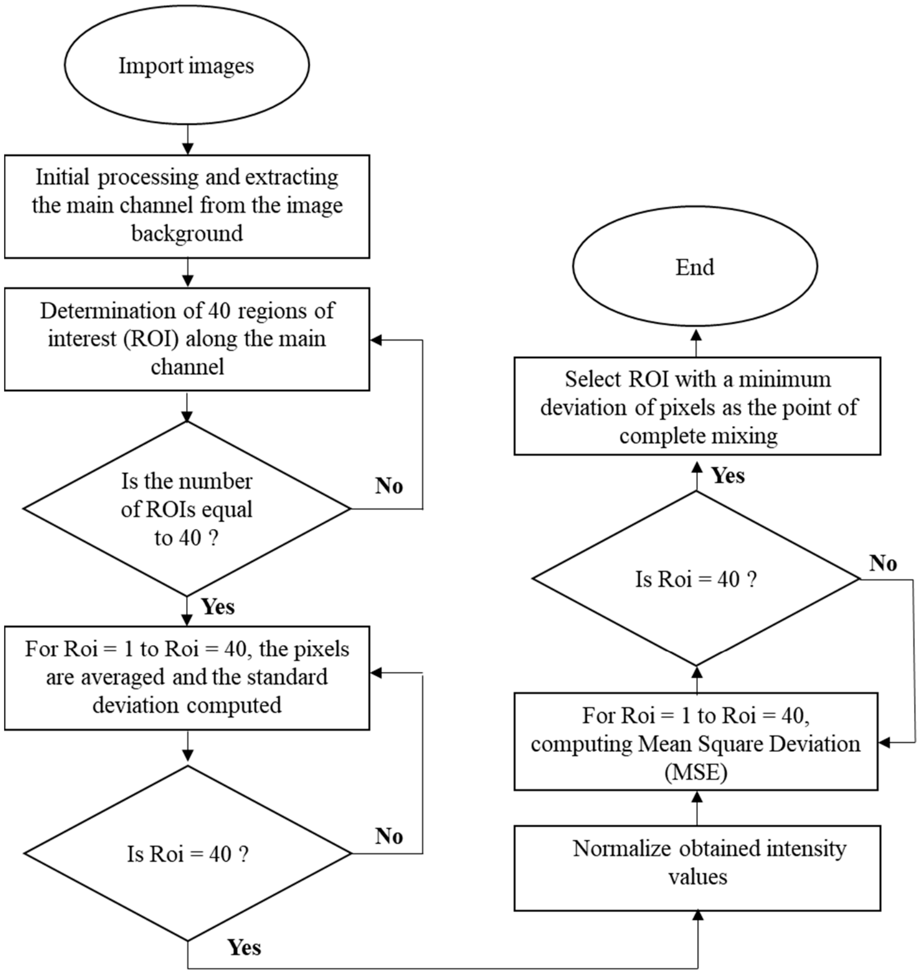

Figure S1. Data flowchart of the colorimetric analysis.

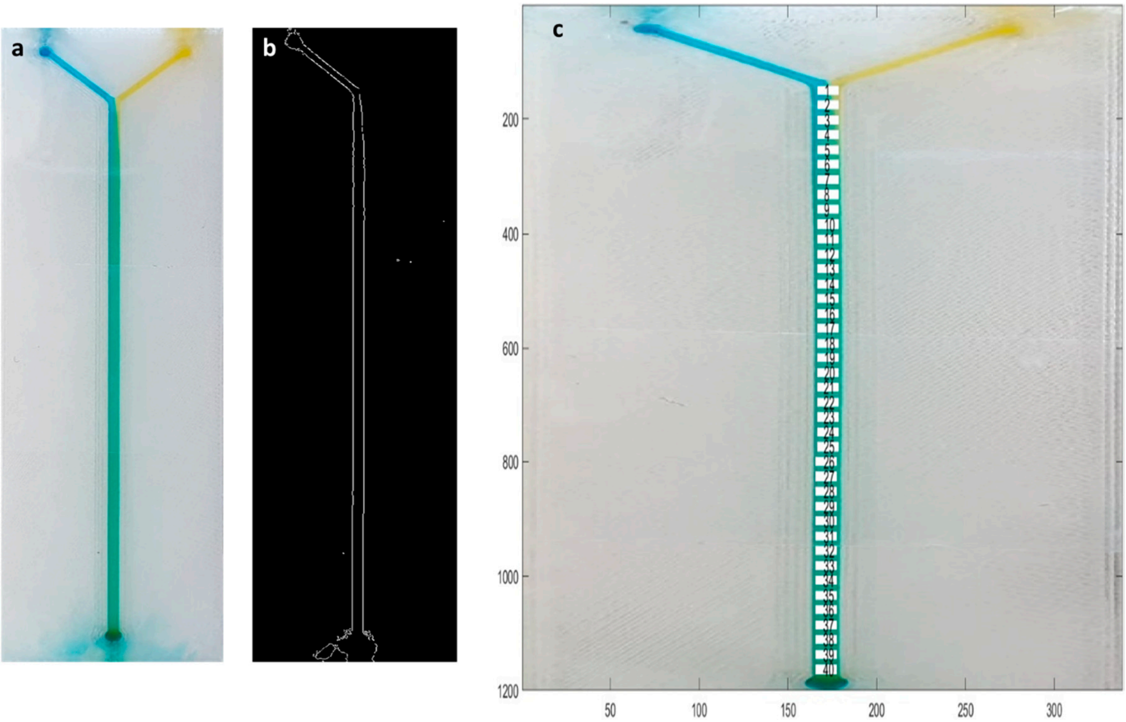

Figure S2. (a) Captured image from the top of the device; (b) detection of the main channel from the input image; (c) different ROIs along the main channel at regular intervals.

Figure 7 describes the normalized color intensity across the channel for 40 ROIs, illustrating the homogenization of the color intensity at the outlet, where the blue stream dissolved in the yellow stream, becoming a homogeneous green color. To find the length of the channel in which complete mixing is achieved, the mean squared deviation was calculated as follows:

$$\mathrm{MSE} = \frac{\sum_{i=1}^{n}(I_i - \bar{I})^2}{n} \tag{2}$$

where I_i denotes the intensity of the i-th pixel in every ROI; n is the total number of pixels in ROI; and $\bar{I}$ is mean intensity, calculated as follows:

$$\bar{I} = \frac{\sum_{i=1}^{n} I_i}{n} \tag{3}$$

The ROI with a minimum deviation of pixels is selected as the point with complete mixing, where at least 90% of the fluids are mixed (Figure 8).

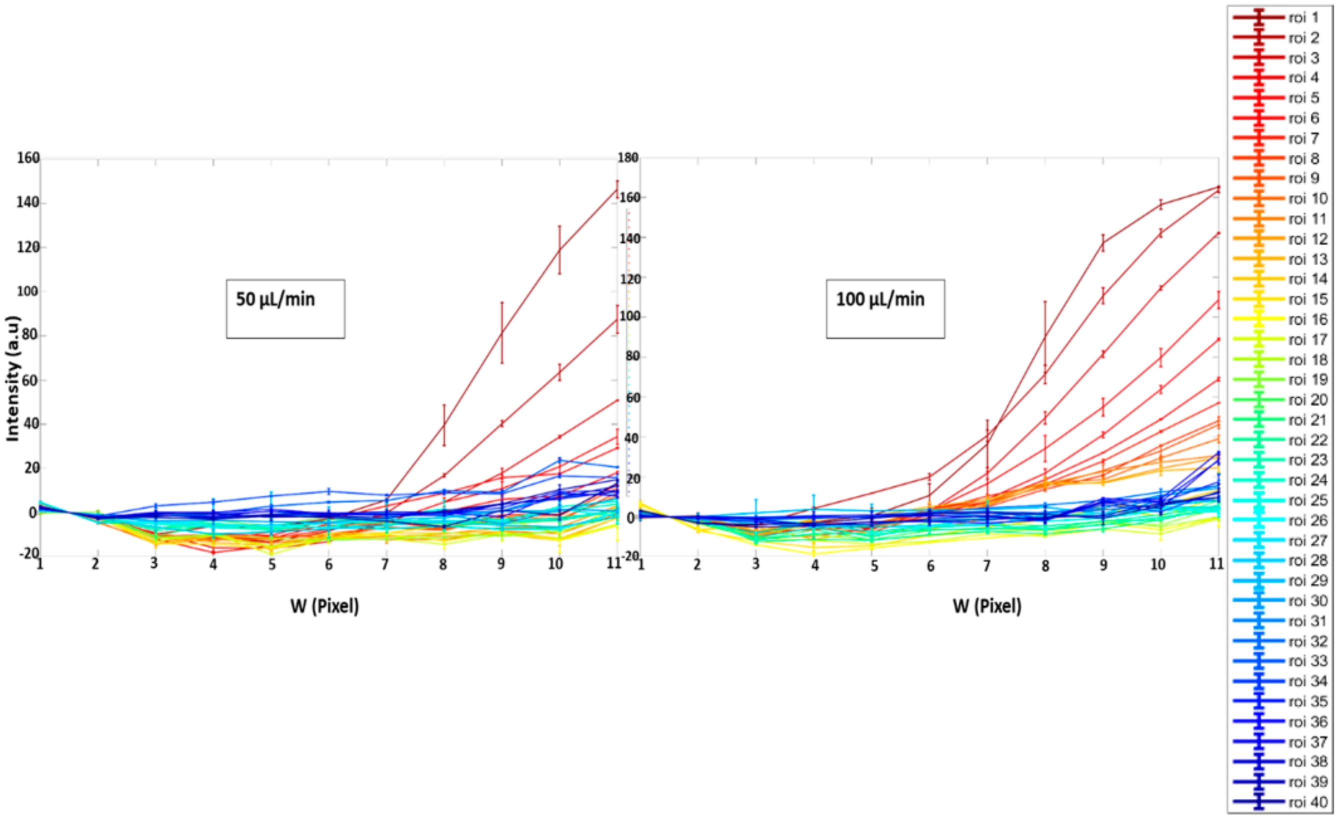

Figure S3. Mixing trend diagrams at flow rates of 50 and 100 µL/min. At the beginning of the main channel, as the fluids are not yet mixed, the curves have two peaks. The curve corresponding to the ROI with complete mixing is a straight line.

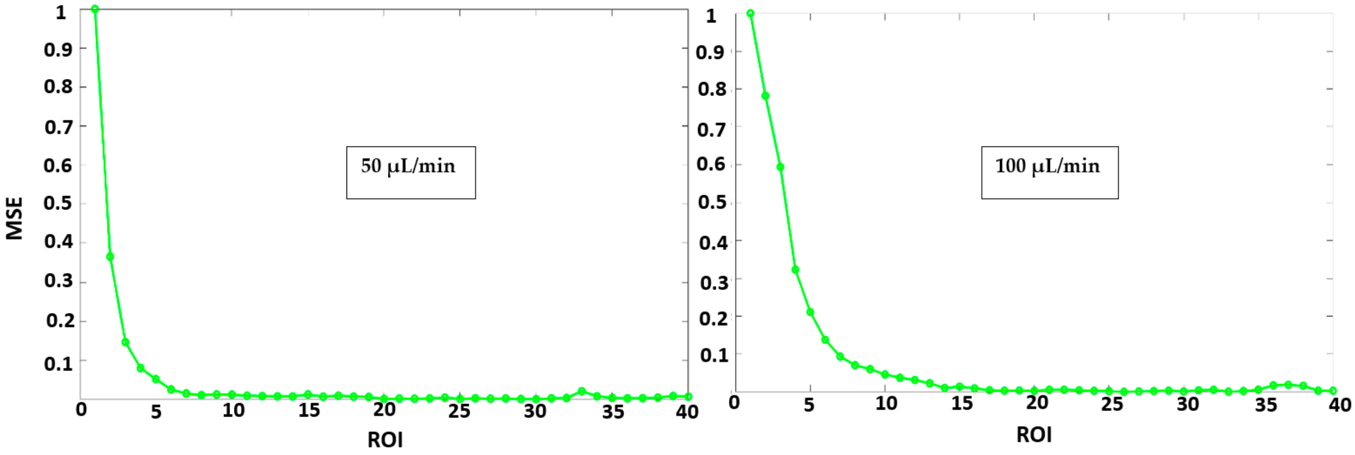

Figure S4. Normalized mean square errors (MSEs) versus different ROIs at flow rates of 50 and 100 µL/min. Complete mixing is achieved after ROI number 7 and 16 at flow rates of 50 and 100 µL/min, respectively.
